# The Effect of Sevelamer on Serum Levels of Gut-Derived Uremic Toxins: Results from In Vitro Experiments and A Multicenter, Double-Blind, Placebo-Controlled, Randomized Clinical Trial

**DOI:** 10.3390/toxins11050279

**Published:** 2019-05-17

**Authors:** Youssef Bennis, Yan Cluet, Dimitri Titeca-Beauport, Najeh El Esper, Pablo Ureña, Sandra Bodeau, Christian Combe, Bertrand Dussol, Denis Fouque, Gabriel Choukroun, Sophie Liabeuf

**Affiliations:** 1 Pharmacology Department, Amiens University Hospital, 80000 Amiens, France; bennis.youssef@chu-amiens.fr (Y.B.); cluet.yan@gmail.com (Y.C.); Bodeau.Sandra@chu-amiens.fr (S.B.); 2 MP3CV Laboratory, EA7517, University of Picardie Jules Verne, 80000 Amiens, France; Titeca.Dimitri@chu-amiens.fr (D.T.-B.); choukroun.gabriel@chu-amiens.fr (G.C.); 3 Nephrology Department, Amiens University Hospital, 80000 Amiens, France; elesper.najeh@chu-amiens.fr; 4 Department of Nephrology and Dialysis, AURA Nord Saint Ouen, 93400 Saint Ouen, France; urena.pablo@wanadoo.fr; 5 Nephrology Department, Bordeaux University Hospital, 33000 Bordeaux, France; christian.combe@chu-bordeaux.fr; 6 Clinical Inverstigation Center, Aix Marseille University, 13354 Marseille, France; bertrand.dussol@ap-hm.fr; 7 Dept Nephrology, Université de Lyon, Hospital Lyon Sud, F-69495 Pierre-Benite, France; denis.fouque@univ-lyon1.fr

**Keywords:** sevelamer, uremic toxins

## Abstract

High serum levels of gut-derived uremic toxins, especially p-cresyl sulfate (pCS), indoxyl sulfate (IS) and indole acetic acid (IAA), have been linked to adverse outcomes in patients with chronic kidney disease (CKD). Sevelamer carbonate could represent an interesting option to limit the elevation of gut-derived uremic toxins. The aim of the present study was to evaluate the adsorptive effect of sevelamer carbonate on different gut-derived protein-bound uremic toxins or their precursors in vitro, and its impact on the serum levels of pCS, IS and IAA in patients with CKD stage 3b/4. For the in vitro experiments, IAA, p-cresol (precursor of pCS) and indole (precursor of IS), each at a final concentration of 1 or 10 µg/mL, were incubated in centrifugal 30 kDa filter devices with 3 or 15 mg/mL sevelamer carbonate in phosphate-buffered saline at a pH adjusted to 6 or 8. Then, samples were centrifuged and free uremic toxins in the filtrates were analyzed. As a control experiment, the adsorption of phosphate was also evaluated. Additionally, patients with stage 3b/4 CKD (defined as an eGFR between 15 and 45 mL/min per 1.73 m^2^) were included in a multicenter, double-blind, placebo-controlled, randomized clinical trial. The participants received either placebo or sevelamer carbonate (4.8 g) three times a day for 12 weeks. The concentrations of the toxins and their precursors were measured using a validated high-performance liquid chromatography method with a diode array detector. In vitro, regardless of the pH and concentration tested, sevelamer carbonate did not show adsorption of indole and p-cresol. Conversely, with 10 µg/mL IAA, use of a high concentration of sevelamer carbonate (15 mg/mL) resulted in a significant toxin adsorption both at pH 8 (mean reduction: 26.3 ± 3.4%) and pH 6 (mean reduction: 38.7 ± 1.7%). In patients with CKD stage 3b/4, a 12-week course of treatment with sevelamer carbonate was not associated with significant decreases in serum pCS, IS and IAA levels (median difference to baseline levels: −0.12, 0.26 and −0.06 µg/mL in the sevelamer group vs. 1.97, 0.38 and 0.05 µg/mL in the placebo group, respectively). Finally, in vitro, sevelamer carbonate was capable of chelating a gut-derived uremic toxin IAA but not p-cresol and indole, the precursors of pCS and IS in the gut. In a well-designed clinical study of patients with stage 3b/4 CKD, a 12-week course of treatment with sevelamer carbonate was not associated with significant changes in the serum concentrations of pCS, IS and IAA.

## 1. Introduction

Patients with late-stage chronic kidney disease (CKD) are permanently exposed to uremic toxins as a result of progressive nephron loss and dietary protein metabolites. These toxins also have a role in the development of uremia-related complications, such as cardiovascular and brain disorders. Uremic toxins can be classified into three classes as a function of their molecular weight: Small, water-soluble molecules (e.g., urea and creatinine), middle molecules (e.g., beta-2-microglobulin) and protein-bound uremic toxins [1]. Alternatively, toxins can be classified according to their origin (microbiota-derived or not) [2]. Indeed, the uremic toxins indoxyl sulfate (IS), indole acetic acid (IAA) and p-cresyl sulfate (pCS) emanate from the gut microbiota’s metabolism of indole and p-cresol that are derived from tryptophan and tyrosine respectively. While IS and pCS are liver metabolites, IAA can be produced directly in the intestine before being absorbed [3]. IS, IAA and pCS are mainly cleared from the body by urinary excretion, but both biliary excretion and intestine secretion are possible and can be strengthened in the case of renal deficiency [4,5,6]. Moreover, the most part of p-cresol metabolites (and other phenolic compounds) excreted in the bile is known to undergo enterohepatic circulation [7].

These microbiota-derived compounds have emerged as a major subclass of protein-bound uremic toxins; they are thought to have a negative impact on the kidney, insulin resistance and might also be involved in the pathogenesis of vascular disease [8]. Indeed, high circulating levels of IS, IAA and pCS have been linked to an elevated incidence of cardiovascular events [9,10,11,12]. Due to the potential toxicity of high levels of uremic toxins, preventing the latter’s accumulation might be a promising way of reducing the complications associated with CKD. This could potentially be achieved by reducing the production of toxins and/or by accelerating their elimination [13]. In Japan, the orally administered charcoal adsorbent AST-120 (Kremezin, Kureha Chemical Industry, Tokyo, Japan) has been used to adsorb uremic toxins [14], although its clinical impact requires further evaluation. The poly(allylamine) phosphate binder sevelamer carbonate is used to limit hyperphosphatemia in CKD patients. When taken with meals, the polymer binds dietary phosphate and prevents its absorption. Sevelamer carbonate is not absorbed from the gut, and thus has a solely local action. It is partially ionized in the bowel, and interacts with phosphate through ionic and hydrogen bonds. When bound to phosphate, other molecules (including uric acid [15], bile acids [16], vitamin C, vitamin K, folic acid [17], ciprofloxacin [18] and levothyroxine [19]) can also be adsorbed by sevelamer. Given the presence of indole, cresol and IAA in the gut, in the case of CKD, sevelamer may therefore constitute a valuable means of limiting the accumulation of gut-derived compounds. Only one abstract publication has reported on sevelamer hydrochloride’s ability to adsorb the uremic toxins IS, indole and p-cresol in the intestinal lumen [20]. However, the few preclinical and clinical studies of sevelamer’s effect on gut-derived uremic toxins has given inconclusive, conflicting results [20,21,22]. Furthermore, Brandenburg et al. observed a significant rise in serum p-cresol levels in dialyzed patients treated with the sevelamer group [21]. Given that these data have not been confirmed, we decided to evaluate sevelamer’s effect on uremic toxins in pre-dialysis patients (i.e., when toxins levels were lower). 

Hence, the objective of the present study was to evaluate i) the adsorption effect in vitro of sevelamer carbonate on one gut-derived protein-bound uremic toxins IAA and on the precursor of pCS named p-cresol and precursor of IS named indole and ii) the impact of sevelamer carbonate on uremic toxins levels in the serum of patients with CKD included in a multicenter, double-blind, placebo-controlled, parallel-group, randomized clinical trial.

## 2. Results

### 2.1. The Impact of Sevelamer In Vitro

In the absence of sevelamer (and regardless of the pH and concentration tested), there were no significant differences in the concentration of p-Cresol, Indole and IAA in filtered versus non-filtered samples (Supplementary Figure S1).

After incubation with sevelamer, a significant decrease (relative to a control without sevelamer) in the filtrates’ phosphorus concentration was observed at both pH 8 (mean ± SD reduction: 20.7 ± 7.6% with 3 mg/mL sevelamer, and 74 ± 3.5% with 15 mg/mL sevelamer) and pH 6 (mean reduction: 78.7 ± 2.4% with 3 mg/mL, and 93.7 ± 0.7% with 15 mg/mL) (Supplementary Figure S2). 

**Figure 1:**
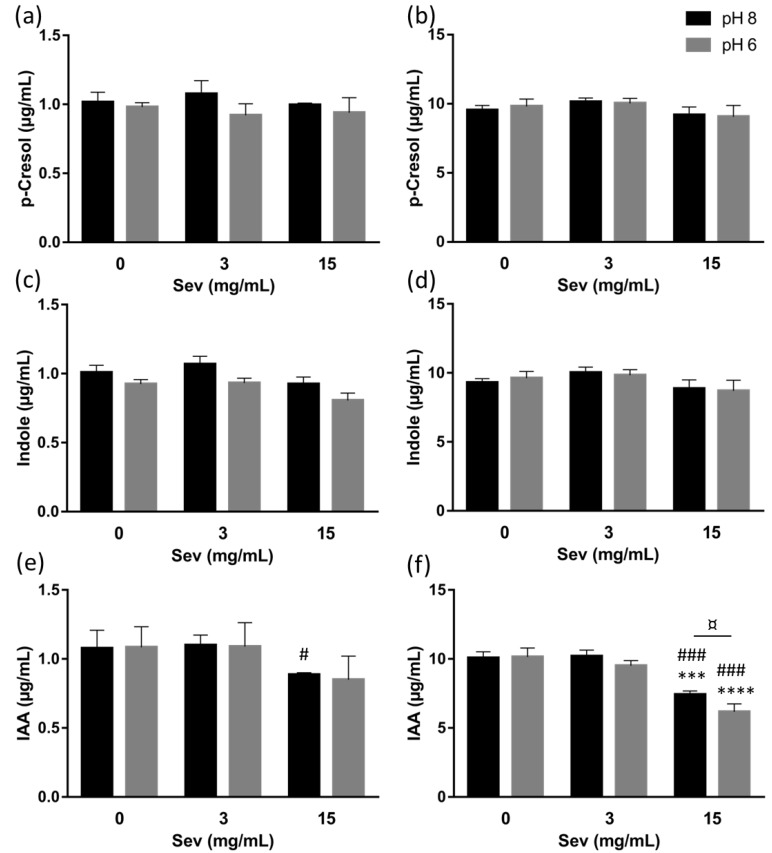
Sevelamer carbonate does not adsorb p-Cresol and Indole but significantly adsorbs IAA at a high concentration. Concentration in the filtrates of p-cresol (**a**,**b**); indole (**c**,**d**) and IAA (**e**,**f**) with 0, 3 or 15 mg/mL sevelamer carbonate, at pH 8 or 6, with 1 µg/mL (a, c and e) or 10 µg/mL (b, d and f) initial concentration. Data are expressed as the mean ± SD (*n* = 3–4). *** *p* < 0.001 vs. Sev 0 mg/mL same pH condition, **** *p* < 0.0001 vs. Sev 0 mg/mL same pH condition, # *p* < 0.05 vs. Sev 3 mg/mL same pH condition, ### *p* < 0.0001 vs. Sev 3 mg/mL same pH condition, ¤ *p* < 0.05. Abbreviations: IAA, indole acid acetic; Sev, Sevelamer.

With regard to the precursors, neither 3 nor 15 mg/mL sevelamer had any effect on the filtrate concentration of p-cresol (Figure 1a,b) or indole (Figure 1c,d) at either of the initial precursor concentrations (1 or 10 µg/mL) and at pH 8 or pH 6. With regard to IAA, 3 mg/mL of sevelamer did not show a significant effect on the filtrate concentration of IAA (Figure 1e,f) at either of the initial toxin concentrations (1 or 10 µg/mL) and at pH 8 or pH 6. With 15 mg/mL of sevelamer and an initial toxin concentration of 1 µg/mL, there were low reductions (mean reduction: 11.5 ± 1.6% at pH 8 and 15 ± 17% at pH 6) in the initial concentration of IAA (Figure 1e). Conversely, with 15 mg/mL of sevelamer and an initial toxin concentration of 10 µg/mL, the concentration of IAA (Figure 1f) in the filtrates was significantly reduced (relative to a control without sevelamer) at both pH 8 (mean reduction: 26.3 ± 3.4%) and pH 6 (mean reduction: 38.7 ± 1.7%). At a starting concentration of 10 µg/mL, IAA was adsorbed more significantly by 15 mg/mL of sevelamer than by 3 mg/mL of sevelamer (Figure 1f), at both pH 8 (mean reduction: 27.2 ± 4.2%, respectively, relative to 3 mg/mL of sevelamer) and pH 6 (mean reduction: 34.2 ± 5.1% respectively, relative to 3 mg/mL sevelamer). In addition, the incubation of 10 µg/mL IAA with 15 mg/mL of sevelamer was associated with significantly higher adsorption at pH 6 than at pH 8 (mean reduction, relative to control without sevelamer: 38.7 ± 1.7% at pH 6, and 26.3 ± 3.4% at pH 8; Figure 1f).

### 2.2. Impact of Sevelamer in Chronic Kidney Disease Patients

The baseline characteristics of randomized patients is summarized in Table 1; the sevelamer and placebo groups did not differ significantly. The mean ± SD age was 63 ± 13, 71% of the participants were male, the mean serum phosphorus level was 1.24 ± 0.17 mmol/L, and the mean eGFR was 27 ± 9 mL/min per 1.73 m^2^. The median (IQR) toxin level was 3.57 (1.32; 6.31) µg/mL for IS, 10.65 (6.05; 17.69) µg/mL for pCS and 1.02 (0.58; 1.44) µg/mL for IAA.

As reported in the original article, there were no statistically significant differences between the sevelamer and placebo group in the changes in serum levels of C-terminal FGF23, intact FGF23, α-klotho, and phosphate [24]. However, the urinary phosphate-to-creatinine ratio and total and LDL cholesterol levels fell significantly in the sevelamer group but remained stable in the placebo group. In the intention-to-treat population, the mean adherence rate was 88% in the placebo group and 86% in the sevelamer group [24].

**Figure 2:**
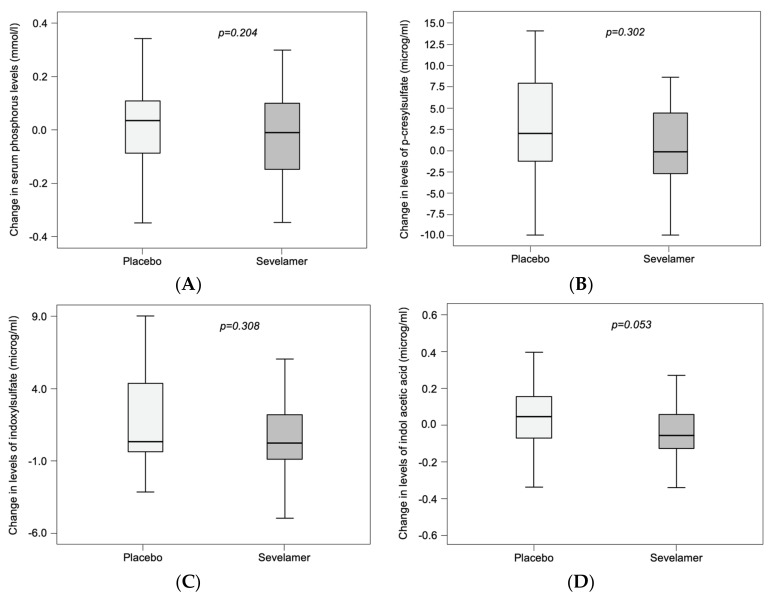
The absence of a significant sevelamer versus placebo difference in serum levels of phosphate and uremic toxins. Changes in serum levels of (**A**) phosphate, (**B**) p-cresylsulfate, (**C**) indoxyl sulfate and (**D**) serum indole acetic acid. Dots in Figure 2B,D represents outlier patients.

Seventy two of the 78 randomized patients had a complete set of assay data for IAA, IS and pCS at inclusion and at the end-of-study visit. After 12 weeks of treatment, the placebo and sevelamer groups did not differ significantly (after correction for the eGFR) in the median change in serum levels of phosphorus (*p* = 0.204), pCS (*p* = 0.302), IS (*p* = 0.308) and IAA (*p* = 0.053) (Figure 2 and Table 2). The intergroup difference for IAA was closest to the significance threshold.

## 3. Discussion

Our combination of in vitro experiments and a clinical study was designed to investigate the ability of sevelamer carbonate to adsorb different gut-derived protein bound uremic toxins or their precursors in vitro, and its impact on the serum levels of pCS, IS and IAA in patients with CKD stage 3b/4. Our in vitro results showed that sevelamer carbonate was able to adsorb IAA but not p-Cresol and Indole, the gut precursors of pCS and IS. In a well-designed clinical study of patients with stage 3b/4 CKD, we did not observe a statistically significant sevelamer versus placebo difference in the change in concentrations of these uremic toxins over 12 weeks of treatment. However, there was a non-significant trend for the change in the IAA concentrations.

In the in vitro incubation experiments, we used a filtration device with a 30 kDa pore size; this retained the sevelamer polymer but not the uremic toxins and their precursors. Next, in order to control for a possible effect on pH, we carried out the experiments at pH 6 and pH 8, i.e., the physiological pH values in the intestine [25,26]. As a positive control, we confirmed the ability of a sevelamer carbonate solution to chelate phosphorus, as in the intestine. If we focused on chemical structures of tested molecules to explain in vitro results, IAA has a carboxylic acid function, such as bile acids, which are also chelated by sevelamer [16], however the precursors do not present any type of chemical group that could be chelated by sevelamer. The in vitro effect of sevelamer on certain uremic toxins and their precursors has been reported in abstract form only [21]. Indeed, De Smet et al. studied the effect of 2.5 mg/mL sevelamer hydrochloride on levels of tryptophan, indole, p-cresol, IS and phosphate at pH 4, 7 and 8. They concluded that regardless of the pH value, sevelamer hydrochloride chelated indole, p-cresol, IS, and phosphate [21]. In contrast, we did not observe a significant effect of sevelamer carbonate on indole and p-cresol. However, De Smet et al. used sevelamer hydrochloride (rather than carbonate) and tested its adsorption effect on toxins in a phosphate-less medium, which was not relevant here. Moreover, the indole concentration they tested was 40 to 400-fold greater than those studied here, while their p-cresol concentration was 7 μg/mL, i.e., the same order of magnitude as the concentrations in our experiments (1 and 10 μg/mL) [21]. Under our experimental conditions, IAA was significantly adsorbed by sevelamer at the higher initial toxin concentration (10 μg/mL). Similarly, significant adsorption of IAA was observed with a high concentration of sevelamer carbonate (15 mg/mL). Although the adsorption of phosphorus by sevelamer carbonate was significantly greater at pH 6 than at pH 8, which was consistent with a previous study showing a higher effect of sevelamer on phosphorus adsorption in an acid condition [27,28]. We did not observe other major differences between pH 6 and pH 8 with regard to sevelamer’s adsorption of uremic toxins; indeed the difference was statistically significant but small for IAA.

The serum concentrations of pCS, IS and IAA measured at baseline from our multicenter, interventional, double-blind, placebo-controlled, randomized clinical study of patients with stage 3b/4 CKD were consistent with the values previously reported in CKD stage 4 (pCS: 3.9 µg/mL; IS: 10.1 µg/mL and IAA: 1.0 µg/mL) [9,10,29]. However, this study did not reveal a significant sevelamer versus placebo difference in the change over time in serum concentrations of pCS, IS or IAA as well as phosphate, after 12 weeks of treatment. We hypothesized that the concentrations of toxins at baseline were not high enough for the effect of sevelamer over 1 year to be shown. Particularly, the initial median IAA concentration of 1.08 (IQ, 0.57; 1.44) μg/mL fell by 0.06 (IQ, −0.12, 0.05) μg/mL (i.e., by around 5%). The clinical relevance of this decrease is questionable even though it was nearly statistically significant (*p* = 0.053). Sevelamer’s effect on percentage reduction in serum toxin concentrations and/or its clinical effect might be greater over a longer treatment period and in stage 5 CKD, where the serum IAA concentration can reach nearly 10 μg/mL. However, it is difficult to estimate the intestinal concentration of sevelamer in humans; this is probably an important parameter, given that sevelamer’s effect was concentration-dependent in our in vitro experiments. It should be noted that the study participants showed good adherence to the treatment, as demonstrated by both pill counts and the decrease in LDL cholesterol levels after 12 weeks of sevelamer treatment [24]. Another hypothesis could be that the sevelamer did not work enough, indeed we failed to find a difference in serum level of phosphate between sevelamer and the placebo group. 

In a previous study conducted in uremic apolipoprotein E–deficient mice, the serum concentration of IS and IAA had not significantly decreased after 8 weeks of a sevelamer-containing diet, on the contrary of the serum phosphorus concentration [30]. Additionally, only a few clinical studies have evaluated the impact of phosphate binders on uremic toxins, but the published results appear to be conflicting. In a non-comparative cross-sectional and observational study, Guida et al. measured the serum pCS concentration in 57 patients on peritoneal dialysis [22]. Among the 45 patients with hyperphosphatemia, 29 were treated with sevelamer hydrochloride and 16 received another phosphate binder. The 12 patients with normal phosphatemia were not treated. There was no significant difference in the serum pCS concentration between patients treated with sevelamer and non-treated patients, or between patients treated with sevelamer and those treated with another phosphate binder [22]. Another recent observational, non-comparative study evaluated the changes over time in serum IS and pCS concentrations in hemodialysis patients after 12 weeks of treatment with sevelamer hydrochloride; although the decrease was significant for pCS, only a non-significant trend was seen for IS [31]. However, the study population was very small (*n* = 5 patients). Brandenburg et al. included 57 hemodialysis patients in an interventional, controlled, cross-over study [21]. Forty-one patients completed the study. After 8 weeks of sevelamer hydrochloride treatment, no changes in serum concentrations were observed for IS and IAA; surprisingly, the serum level of pCS increased significantly [21]. It is important to note that these studies included small numbers of dialyzed patients and did not have a parallel-group and randomized designs. Very recently, a single-blind, placebo-controlled randomized clinical trial compared sevelamer with placebo in non-dialyzed patients with regard to plasma levels of p-cresol, which fell significantly in the sevelamer group only [32]. It should be noted that the circulating toxic form is pCS and not p-cresol, which is essentially generated ex vivo by the treatment of phenol-containing blood samples [33].

The present study’s main strength was its comprehensive design; we tested our hypothesis by combining in vitro experiments with observations from a multicenter, double-blind, randomized clinical trial. The limitations were the limited experimental conditions, including different pH, additional uremic toxins concentrations and reaction times. For the clinical study, the main limitation was the absence of data on the study participants’ diet and that, similar to what has been observed with phosphate, uremic toxins serum level as an outcome measure may have been too insensitive to capture changes in net gastrointestinal absorption. Finally, the study power seemed to be sufficient (near 80%) for IAA, which was the toxin with the least variability. However, a lack of power cannot be excluded for the other toxins with higher variability. 

In conclusion, our in vitro results showed that sevelamer carbonate was capable of chelating the gut-derived uremic toxin IAA but not the IS and pCS gut precursors indole and p-cresol. In a well-designed clinical study of patients with stage 3b/4 CKD, a 12-week course of treatment with sevelamer carbonate was not associated with significant changes in the serum concentrations of the three toxins. There was a non-significant trend towards a decrease in the serum IAA concentration. In view of the known associations between uremic toxins and hard outcomes in CKD, further research on methods of decreasing toxin concentrations is essential.

## 4. Methods

### 4.1. The In Vitro Study

#### 4.1.1. Chemicals

Indole, p-cresol, IS and IAA were obtained from Sigma Aldrich (Saint Quentin Fallavier, France), and pCS was purchased from TCI Chemicals Europe (Paris, France). Stock solutions were prepared in methanol (for IAA and p-cresol) or reverse osmosis water (indole) at a concentration of 1 mg/mL and stored at −25 ± 5 °C. Working solutions were prepared by mixing and diluting the stock solutions in phosphate buffered saline (PBS, Sigma Aldrich, Saint Quentin Fallavier, France). Sevelamer carbonate was obtained from 2.4 g packets of Renvela^®^ (Sanofi Aventis, Paris, France). The powder was dissolved in PBS to obtain a 30 mg/mL working solution.

#### 4.1.2. Adsorption Experiments

Samples containing the IAA and two precursors (indole and p-cresol) at a final concentration of 1 or 10 µg/mL in PBS (pH adjusted to 6 or 8, as expected in the gut) were incubated in Amicon^®^ Ultra-0.5 centrifugal 30 kDa filter devices (Merck-Millipore, Molsheim, France) with gentle agitation for 120 min at 37 °C in the presence or absence of sevelamer carbonate. Two concentrations of sevelamer carbonate were tested: 3 mg/mL was close to the concentrations used in literature reports on sevelamer’s adsorption of uric acid [15], bile acids [11], vitamin C, vitamin K, and folic acid [12], and 15 mg/mL was approximately the concentration obtained when 4.8 g of sevelamer carbonate was dispersed in a volume of 320 mL, which corresponded to the average volume of small intestine fluid distributed into 6 “pockets” as measured in healthy volunteers after a meal [24]. Next, the samples were centrifuged at 10,000× *g* for 10 min, and the filtrates were assayed for the uremic toxins. To check that the toxins and precursors were not bound to the filter under our experimental conditions, concentrations of IAA, indole and p-cresol in non-filtered solutions were also measured. Lastly, to confirm that sevelamer was able to adsorb phosphate under our experimental conditions, the inorganic phosphorus concentrations in filtrates after incubation were compared with those in the absence of sevelamer.

#### 4.1.3. Analysis of Uremic Toxin Concentrations in Filtrates

The concentrations of toxins and precursors in filtrates were measured using a validated high-performance liquid chromatography (HPLC) method, with a diode array detector (DAD) set to 270 nm (Alliance^®^, Waters, Milford, MA, USA) using a 3 µm 3.9 mm × 150 mm Atlantis dC18 column (Waters, Milford, MA, USA) and a solvent gradient (acetonitrile/0.1 M ammonium formate buffer, pH 3) at a flow rate of 0.8 mL/min. The lower limit of quantification (LOQ) was 0.2 µg/mL for IAA, indole and p-cresol. The method gave linear results between the LOQ and 50 µg/mL (correlation coefficient: >0.999). The intraday and interday coefficients of variation were all below 15%, and the bias ranged from −12.9% to 7.1%. This method was validated according to the European Medicine Agency’s guideline on bioanalytical method validation.

#### 4.1.4. Analysis of The Inorganic Phosphorus Concentration in Filtrates

Inorganic phosphorus was assayed on an RX Daytona+ system (Randox Laboratories, Crumlin, UK), according to the manufacturer’s instructions.

### 4.2. Clinical Study

#### 4.2.1. Study Design

Ninety-six patients with stage 3b/4 CKD (defined as an eGFR between 15 and 45 mL/min per 1.73 m^2^) were included in the “FGF23 Reduction Efficacy of a New Phosphate Binder in Chronic Kidney Disease” (FRENCH) multicenter double-blind, placebo-controlled, parallel-group, randomized clinical trial between December 2010 and December 2012. After screening, 78 patients were randomized by 14 nephrology outpatient clinics in France (Appendix A). The study’s objectives and procedures were approved by an independent investigational review board (CPP Nord Ouest II, Amiens, France; approval date 2 June 2010, ref 2010/19) and the French drug agency (Approval Date 30 July 2010, ref A100615-32). Each patient provided written informed consent to participate in the study. The study was registered at clinicaltrials.gov (NCT01220843) in October 2010. Details of the study population and the study design have been published elsewhere [25].

The FRENCH study’s primary objective was to assess sevelamer carbonate’s effect (relative to that of placebo) on the serum C-terminal FGF23 levels. The present report describes one of the study’s secondary objectives: The evaluation of sevelamer carbonate’s effect (relative to a placebo) on uremic toxin levels.

#### 4.2.2. Sample Size Determination

The sample size of 80 patients was initially calculated on the FRENCH study’s primary objective to detect a 30% reduction in median C-terminal FGF23 levels in the sevelamer carbonate group compared with the placebo group after 3 months [25]. Evaluation of the reduction in pCS, IS and IAA levels in the sevelamer carbonate group compared with the placebo group was a secondary objective of the FRENCH study and did not drive the sample size determination. However, the study power seemed to be sufficient (near 80%) for the detection of IAA (toxin with the least variability) differences.

#### 4.2.3. Study Procedures

After a screening visit, participants completed a 1- to 2-week run-in period during which baseline blood and urine samples were collected. This was followed by a 12-week treatment period. At randomization, the study personnel and participants were blinded to the use of phosphate binder. The study participants took a dose of 4.8 g of sevelamer or placebo three times a day for 12 weeks. If the serum phosphate level fell below 0.8 mmol/L, the dose of treatment was halved. Adherence to study medication was monitored via pill counts.

#### 4.2.4. Clinical and Laboratory Evaluations

All patients had an interview with a physician (to establish their personal medical history) and underwent a clinical examination. Age, gender, anthropometric data, and medication use were recorded. Fasting blood samples were collected after inclusion in the study and after 12 weeks of treatment.

#### 4.2.5. Analysis of Uremic Toxins in Serum Samples

Proteins in standards, quality controls, and patient samples (200 µL of serum, in all cases) were precipitated with 400 µL methanol containing ethofylline as an internal standard. After centrifugation at 14,000× *g* for 10 min, 500 µL of supernatant was evaporated at 40 °C under nitrogen. Next, the dry residues were dissolved in 100 µL of pH 3 0.1 M ammonium formate buffer. Samples were assayed for pCS, IS and IAA using the validated HPLC-DAD method described above.

### 4.3. Statistical Analysis 

In the in vitro study, intergroup comparisons were performed using an analysis of variance (for normally distributed data) or a Kruskal–Wallis test (for non-normally distributed data), followed respectively by a Tukey post hoc test or a non-corrected Dunnett’s post hoc test.

In the clinical study, all patients with available data were analyzed. Data were expressed as the mean ± standard deviation (SD), median (interquartile range [IQR], or the number (percentage)), as appropriate. Patients were stratified by treatment group. Intergroup comparisons were performed with a chi-squared test (for categorical variables) or a t test or Mann–Whitney test (for continuous variables). We compared the changes in phosphorus and uremic toxin levels over time (i.e., between randomization and week 12) in the two treatment groups. The paired *t* test method was applied for the comparison of repeated measures. 

The threshold for statistical significance was set to *p* ≤ 0.05. All statistical analyses were performed using SPSS software (version 18.0, SPSS Inc., Chicago, IL, USA) for Windows (Microsoft Corp, Redmond, WA, USA) or GraphPad Prism (version 7.0, GraphPad Software Inc., San Diego, CA, USA).

## Figures and Tables

**Table 1:** Baseline characteristics of the randomized study participants.

Variables	All *n* = 78	Placebo *n* = 39	Sevelamer *n* = 39	*p*
Age (years)	63 ± 13	63 ± 14	63 ± 13	0.918
Male gender, *n* (%)	55 (70.5%)	28 (71.8%)	27 (69.2%)	0.804
GFR MDRD (mL/min per 1.73 m^2^)	27.0 ± 9.1	28.1 ± 9.9	25.4 ± 8.1	0.193
Serum phosphorus (mmol/L)	1.24 ± 0.17	1.25 ± 0.16	1.23 ± 0.18	0.705
Serum calcium (mmol/L)	2.34 ± 0.11	2.33 ± 0.11	2.35 ± 0.11	0.486
Serum creatinine (µmol/L)	234 ± 76	227 ± 75	241 ± 79	0.379
Serum albumin (g/L)	41.7 ± 3.4	41.5 ± 3.7	41.9 ± 3.1	0.645
Serum intact PTH (pg/mL)	97 (61; 127)	103 (71; 141)	91 (58; 123)	0.169
Serum total cholesterol (mmol/L)	4.7 ± 1.1	4.7 ± 1.0	4.7 ± 1.2	0.522
Serum LDL cholesterol (mmol/L)	2.5 ± 1.0	2.4 ± 1.0	2.6 ± 1.0	0.430
Serum indoxyl sulfate (µg/mL) * 0.53 ± 0.29 µg/mL in healthy adults ^#^ [23]	3.57 (1.32; 6.31)	3.45 (1.07; 5.61)	4.26 (1.77; 7.35)	0.119
Serum p-cresyl sulfate (µg/mL) * 1.9 ± 1.3 µg/mL in healthy adults ^#^ [23]	10.65 (6.05; 17.69)	10.34 (5.12; 21.00)	11.34 (7.82; 16.44)	0.681
Serum indole acetic acid (µg/mL) * 0.5 ± 0.3 µg/mL in healthy adults ^#^ [23]	1.02 (0.58; 1.44)	0.96 (0.59; 1.47)	1.08 (0.57; 1.44)	0.812

Data are quoted as the mean ± SD for normally distributed variables, the median (interquartile range) for non-normally distributed variables, or the number (percentage) for qualitative variables. GFR MDRD: Glomerular filtration rate estimated using the Modification of Diet in Renal Disease equation; FGF23, fibroblast growth factor 23; RU, relative units; PTH, parathyroid hormone. * Available for 72 patients (placebo group: *n* = 35; sevelamer group: *n* = 37). # [23].

**Table 2:** Change over time in clinical biochemistry parameters during the study period.

Toxin	Visit 1 (V1)	Visit 5 (V5)	V5-V1
**Phosphorus (mmol/L)**			
Placebo	1.25 ± 0.16	1.26 ± 0.19	0.01 ± 0.16
Sevelamer	1.23 ± 0.18	1.20 ± 0.19	−0.04 ± 0.20
**pCS (µg/mL)**			
Placebo	10.34 (5.12; 21.00)	11.69 (7.97; 25.37)	1.97 (−1.33; 7.89)
Sevelamer	11.34 (7.82; 16.44)	11.17 (8.89; 16.13)	−0.12 (−2.70; 4.30)
**IS (µg/mL)**			
Placebo	3.45 (1.07; 5.61)	3.46 (1.65; 9.99)	0.38 (−0.36; 4.39)
Sevelamer	4.26 (1.77; 7.35)	3.84 (2.10; 7.49)	0.26 (−0.85; 2.20)
**IAA (µg/mL)**			
Placebo	0.96 (0.59; 1.47)	0.99 (0.58; 1.65)	0.05 (−0.07; 0.16)
Sevelamer	1.08 (0.57; 1.44)	0.92 (0.57; 1.44)	−0.06 (−0.12; 0.05)

Data are quoted as the mean ± SD for normally distributed variables, and the median (interquartile range) for non-normally distributed variables. pCS, p-cresyl sulfate; IS, indoxyl sulfate; IAA, indole acetic acid.

## Supplementary Materials

The following are available online at https://www.mdpi.com/2072-6651/11/5/279/s1, Figure S1: In the absence of sevelamer carbonate, the filter devices did not retain the toxins, Figure S2: Sevelamer carbonate effectively adsorbs phosphorus.

## Appendix A

National coordination: Amiens University Hospital: Prof. Gabriel Choukroun (lead investigator, study coordinator), Dr Sophie Liabeuf, Dr Najeh El Esper, Dr Maité Jaureguy, Dr Caroline Lecaque, Prof. Ziad Massy, Prof. Jean-Marc Chillon, Dr Romuald Mentaverri, Dr Momar Diouf, Carl Picard, Jonathan Meynier, Marie-Laurence Lepilliez.

Local clinical centers: Bordeaux: Pr Christian Combe, Marine Douillet; Caen: Pr Jean-Philippe Ryckelynck, Mohamed Ouethrani; Marseille: Pr Bertrand Dussol, Laurent Samson; Saint Ouen: Pr Pablo Urena; Nancy: Pr Luc Frimat; Paris Necker: Pr Dominique Prié; Paris Pompidou: Pr Pascal Houllier, Pr Eric Thervet; Paris Tenon: Dr Courbebaisse; Saint Etienne: Dr Eric Alamartine; Nice: Pr Vincent Esnault, Dr Guillaume Alexandre Favre; Montpellier: Pr Bernard Canaud, Dr Leila Chenine Khoualef, Dr Hélène Leray Moragues, Dr Marion Morena; Lyon: Pr Denis Fouque; Lyon Tassin: Pr Guillaume Jean; Valenciennes: Pr Philippe Vanhille.
