# Supplementary material for: The Effect of Sevelamer on Serum Levels of Gut-Derived Uremic Toxins: Results from In Vitro Experiments and A Multicenter, Double-Blind, Placebo-Controlled, Randomized Clinical Trial
Source: Toxins (Basel). 2019 May 17;11(5):279. doi: 10.3390/toxins11050279 (PMC6563242; doi:10.3390/toxins11050279)
Supplement: Supplementary file 1 [file toxins-11-00279-s001.pdf]

Supplementary Materials: The Effect of Sevelamer on Serum Levels of Gut-Derived Uremic Toxins: Results from in vitro Experiments and A Multicenter, Double-Blind, Placebo-Controlled, Randomized Clinical Trial

Bennis Youssef, Cluet Yan, Titeca-Beauport Dimitri, El Esper Najeh, Pablo Ureña, Bodeau Sandra, Combe Christian, Dussol Bertrand, Fouque Denis, Choukroun Gabriel and Liabeuf Sophie

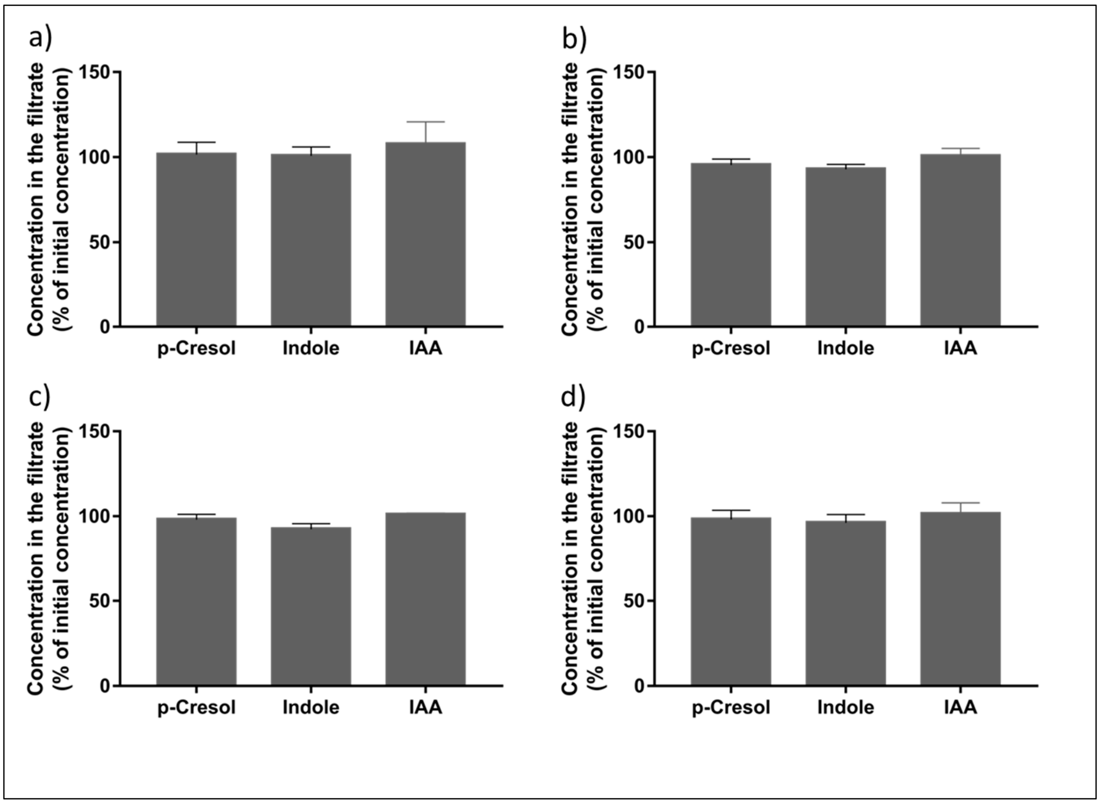

Figure S1. In the absence of sevelamer carbonate, the filter devices did not retain the toxins. The concentrations of indoxyl sulfate (IS) and p-cresyl sulfate (pCS) gut precursors (p-Cresol and Indole) and IAA in the filtrates were measured at pH 8 (**a**,**b**) and pH 6 (**c**,**d**) after centrifugation of samples, with initial concentrations of 1 μg/mL (a, c) and 10 μg/mL (b, d). Data are expressed as the mean ± SD value relative to the initial concentration (i.e. 100%) before centrifugation (n = 3–4). Abbreviation: IAA, indole acetic acid.

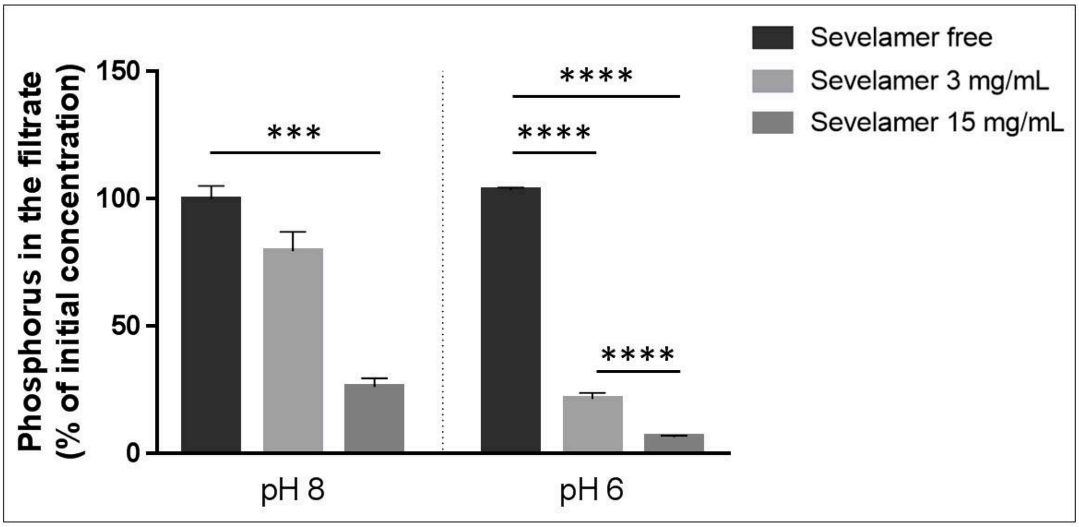

Figure S2. Sevelamer carbonate effectively adsorbs phosphorus. Phosphorus concentration in the filtrate after incubation with 0, 3 or 15 mg/mL sevelamer carbonate at pH 8 or 6. Data are expressed as the mean ± SD, relative to the initial concentration before centrifugation (n = 4–6). *** $p < 0.001$, **** $p < 0.0001$.
